# A Lecture on Epistaxis, Vel Hæmorrhagia Nasi

**Published:** 1839-02-23

**Authors:** N. Chapman

**Affiliations:** Professor of the Theory and Practice of Physic, in the University of Pennsylvania


### MEDICAL EXAMINER.

DEVOTED TO MEDICINE, SURGERY, AND THE COLLATERAL SCIENCES.

No. 8.] PHILADELPHIA, SATURDAY, FEBRUARY 23, 1839. [Vol. II.

### A LECTURE ON EPISTAXIS, VEL HÆMORRHAGIA NASI.

*By* N. Chapman, M. D., *Professor of the Theory and Practice of Physic, in the University of Pennsylvania.*

[Reported for this Journal.]

As unhappily has the first of these titles, signifying distillation, been selected as most others, to signify the varieties of hæmorrhage, it really having no meaning in its present application. The second, *hæmorrhagia nasi*, expresses precisely, what is desired to be conveyed, and should be adopted, to the exclusion of the ancient term.

Bleedings from the nose may be, as the other hæmorrhages, either active or otherwise. The first state is often preceded by evidence of undue determination of blood to the head, as a sense of fulness, or tensive pain, vertigo, tinnitus auriam, or other noises, flashes of light before the eyes, which are sometimes injected and red, a flushed, tumid countenance, heat and itching in the nostrils, with even a slight degree of swelling, attended by throbbing of the carotid and temporal arteries, and activity of pulse. It sometimes puts on a more distinct febrile character, and here, previously to an attack, there is a cold fit succeeded by fever, or only alternate chills and flushes, observing in the return of the paroxysms, with more or less precision, the order of regular intermittents,—while, in other instances, it comes on without any premonition whatever, a gush of blood following the slightest exertion, or any excitement.

As the vessels spread over the Schneiderian membrane are exceedingly numerous, forming a complete reticulated texture, with a thin and delicate covering, and very much exposed, we are, at all times, peculiarly liable to this hæmorrhage, but it is most apt to happen at an early age, again towards maturity, and on the decline of life. Menstruation occurring, a new train of action is established, and it is comparatively seldom met with in women, where this function is uninterruptedly performed.

Distinct from the period of life, which certainly has an influence, the predisposition to epistaxis chiefly consists in a certain conformation, as the short neck and large head, by which blood is disproportionately invited to the part. It takes place, however, under very different circumstances.* Commonly to be met with in the full or plethoric, the opposite condition is not exempt from its occurrences, provided there are irregularities in the circulation, with special directions of blood to the head. Numerous circumstances conduce to the latter effect, among which are violent exercises, certain efforts in a bent position, straining at stool, also loud speaking or singing, or sneezing or coughing, playing on wind instruments, exposure to intense heat, or the reverse, cold, and especially cold feet, stimulating ingesta, constipated bowels, tight lacing, or cravat. Moreover, it is occasioned by mental emotions, rage or terror, or a very excited imagination, or intense study, or anxiety, with insomnolency. Even to the most trivial causes, is it sometimes to be traced. Thus, Bruyerin gives an example in which the nostrils flowed with blood upon smelling at an apple—Rhodius upon smelling a rose—and Blanchard upon the ringing of bells.

As a secondary affection, epistaxis frequently occurs from cerebral fever, of high excitement, as well as other diseases, acute or chronic, among which visceral obstructions are very apt to induce it, and, I may add, that it is often consequent on suppression of the catamenial and hæmorrhoidal discharges.

Mainly, in these modes, is epistaxis in its several grades of activity produced, and when purely passive, to which state I think it more liable than any hæmorrhage, it must be usually assigned to changes in the blood itself, wrought by those circumstances formerly enumerated, by which the vital powers are impaired, and it rendered more fluid. But what I have said, has reference only to the etiology of epistaxis in its common presentations. As other hæmorrhages, it sometimes prevails so generally as to amount to an epidemic, and to this character, I am inclined to believe, it is particularly disposed. The most remarkable instance, perhaps, of such an occurrence, is to be found in Morgagni, who states that a great mortality took place from it in Tuscany, and other parts of Italy. During the year 1823, hæmorrhage of every description, though mostly from the nose, was observed among us, extremely copious, and difficult of suppression. No satisfactory explanation can be given of these occasional wide-spread prevalences of hæmorrhage. Certainly, they are not owing to the excesses or variations of temperature, or the usual states of the atmosphere. But the latter may undergo some other change at the period, and from the well-known influence of its rarefaction in this respect, such may be that change.

Epistaxis cannot be confounded with any other affection, and the only concern as to the diagnosis, refers to the discrimination of its own varieties, having regard, in the first place, to the mode of its production, and next to its precise character

* Epistaxis is often the result of blows or falls, or other acts of violence. Cases, however, occasioned by accidents, do not come within the definition of vital hæmorrhage.

and the state of system with which it is associated. These are particulars, so easily learnt by an investigation of the case, that the subject may be dismissed without further remark.

Not in excess, the active form of this hæmorrhage is to be deemed salutary, whether it occurs in a general plethoric condition, or in special determinations,—and hence the relief from it, in the excitement of fevers, and, perhaps, in every acute, inflammatory, or active congestive disease. No one, indeed, who has not witnessed it, can well appreciate the effect of the loss of even a few ounces of blood from this source, in the cerebral affections. An explanation, however, of the fact, is afforded in the arrangement of the vessels of the nasal lining. These are supplied chiefly from the internal maxillary artery, which, inosculating freely with some of the ramifications of the internal carotid, blood is diverted from the brain, and its oppressions mitigated or relieved. But the consequence of this hæmorrhage is very much the reverse, when of a less active, and, still more, of a passive nature, or it is postponed to the advanced stage of these affections, having then only a tendency to increase exhaustion, and is, indeed, mostly to be considered of fearful import.

Being active, and original to the part whence the effusion takes place, it is of easy management, and difficult or troublesome, and even dangerous, under opposite circumstances, and the more so, if derived from obstructions or other organic lesions of the thoracic or abdominal viscera.

Confirmed into a habit, it is uniformly to be dreaded. Hippocrates remarks, that thus subjected, young persons are apt to incur diseases of the chest, as pleuritis, pneumonitis, hæmoptysis, and consumption, probably owing to a metastasis of the nasal irritation to the lungs. But such not taking place, it is held to have a contrary effect, or preventive of pulmonary affections.

By the long continuance of it, the system becomes exceedingly deranged, and health impaired in various ways. Even the more recent attacks of it sometimes present the most formidable aspect, proving exceedingly intractable or utterly unmanageable. No hæmorrhage is occasionally more profuse, or in which larger quantities of blood are lost, and among other instances which might be cited to this purport, Bartholin mentions a case of forty-eight pounds—Rhodius another of eighteen pounds, within thirty-six hours, and a respectable writer in the Leipsic Acta Erudita, a third, of not less than seventy-five pounds within ten days. The Ephemera of Natural Curiosities contain a case where the quantity is not stated, from the difficulty of taking an account of it, which continued, without cessation, for six weeks.*

In 1820, I attended an elderly gentleman, who, during a night, must have lost several quarts. He frequently fainted, on which there was uniformly a suspension of the flow, recurring, however, on his revival.

* Good.

Nearly about the same time, one of our most distinguished citizens died of this hæmorrhage, after three weeks' continuance, which the best skill could not control. As well from his general aspect, as that of the blood, which was thin and nearly colourless, he must have become exsanguineous. More recently, I was consulted in the progress of such an attack, in the vicinity of this city, which ended fatally. Cases of this kind usually occur in persons andvanced in life, and of very vitiated habits, having their viscera, the liver or spleen, much disordered. They often prove mortal.

Of the morbid phenomena, on dissection, in epistaxis, I have no knowledge, so far as regards the immediate seat of it, the case not being of sufficient importance to have attracted attention. They may, however, be presumed, from analogy, to be such as are presented in other hæmorrhages of the mucous membrane. Extraneous formations, as polypi, fungoid and other growths, occasionally productive of bleedings, are not properly incidents of spontaneous or vital hæmorrhage. In the secondary forms of the affection, we often discover great disorder of the thoracic and abdominal viscera, the lungs, the heart, the alimentary tube, the liver and spleen especially.

Little need be said of the pathology of epistaxis, it being also, in this respect, analogous to the hæmorrhages of other mucous surfaces, and hence I shall dismiss it with merely suggesting the resemblance between it, in its active form especially, to apoplexy, in the causes and prelusive symptoms, the direction of the extravasation of blood from the nostrils or brain being determined, as it were, accidentally. This is one of the most interesting views in which the affection can be contemplated.

(To be continued.)

